# Analytical and legislative challenges of sewage sludge processing and management

**DOI:** 10.1007/s00706-018-2255-2

**Published:** 2018-08-09

**Authors:** Bartłomiej Michał Cieślik, Lesław Świerczek, Piotr Konieczka

**Affiliations:** Department of Analytical Chemistry, Faculty of Chemistry, Gdańsk University of Technology, Gdańsk, Poland

**Keywords:** Extraction, Ecology, Gas chromatography, High pressure liquid chromatography, Mass spectroscopy, Metals

## Abstract

**Abstract:**

This article presents the most popular methods of sewage sludge management and analytical techniques which could be a powerful tool in designing new sewage sludge management methods. Chemical analysis is also described as a vital point at the subsequent stages of technological processes control and sewage sludge quality assessment. It is also an instrument essential to maintaining control of processed sewage sludge introduced to the environment as ready-to-use materials. The sludge management method is conditioned by the compliance with legal acts concerning sludge management. The most important of these contain information regarding allowable concentrations of pollutants which can be released into the environment, and the most important declarations concerning sewage sludge management. Various analytical techniques and preparation methods that can be used during the monitoring of the managed and processed sewage sludge are described. The most important are chromatographic techniques, methods based on inductively coupled plasma, and mass spectrometry based methods.

**Graphical abstract:**

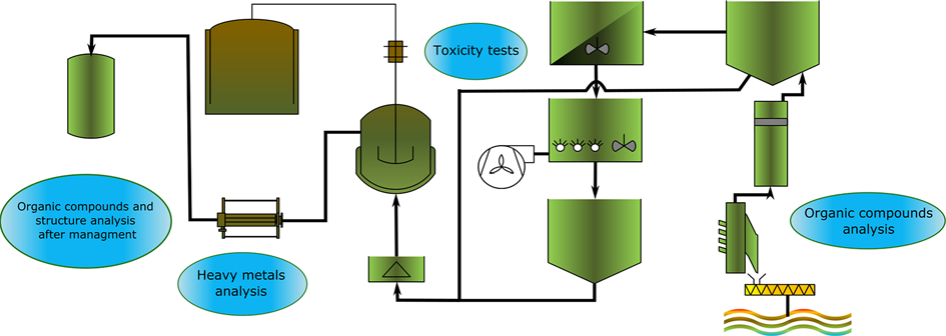

## Introduction

In Europe, management of excess sludge, which is treated as a waste material, is still a problem. Its production increases every year, especially in developing countries. While excess sludge production in Europe has doubled over the last 20 years, in China it has increased twofold in the last 5 years [1–4]. European statistics show that one inhabitant contributes to the production of 90 g of dry mass of excess sludge daily. Since the population of the European Union (EU) exceeded half a billion, sewage sludge production is now above 45,000 tons a day [2]. Therefore, a large number of EU directives concern problems of sludge treatment and waste management. All EU member states are obliged to implement the adopted directives. For this reason, numerous legal acts are issued, which define the highest allowable concentrations of pollutants in the managed materials, leachates and extracts from prepared materials or even individual elements of the environment when releasing various types of waste into it [2]. Each of the possible sludge management methods is characterised by a different level of emissions of various pollutants present in the sludge produced. These parameters depend on:

- the industrialization of the agglomeration where the sludge is collected,
- the size of the agglomeration where the sludge is collected,
- the treatment technology used at the treatment plant,
- the selection of unit processes connected with sludge management.

The selection of the optimal method of excess sludge management is a complicated problem and it depends on two main groups of factors: the kind and level of potentially toxic substances present in the managed sludge and the type and concentration of potentially toxic substances released into the environment after using a given technology. An optimal technology should be a compromise between the possible reduction of the concentration and neutralization of certain groups of hazardous substances [5–8]. One should also consider the possibility of new, potentially hazardous compounds occurring, and an increase in concentrations of those already contained in the managed sludge due to the employed technological processes that lead to the neutralisation of other groups of potentially hazardous substances. It is worth mentioning that the process of wastewater treatment in a wastewater treatment plant (WWTP) is connected with odor emission. This may have a negative influence on public trust and the quality of atmospheric air. Sometimes, despite the need, appropriate deodorization systems are not used at municipal wastewater treatment plants mainly for economic reasons [9].

According to the principles of green chemistry waste should be treated in a holistic manner, so it should be eliminated at the source. The promotion of clean methods and sustainability of resources makes pollution monitoring, both at the stage of wastewater treatment and its management method, a key issue [10]. That is why a vast number of analytical methods are employed to allow the determination of different groups of contaminants at successive stages of sewage sludge management process. Advances in both quantitative and qualitative determinations of various organic compounds have aided the understanding of their occurrence and processes they undergo during wastewater treatment. This is helpful in choosing the right management technique and improving the safety of the aquatic environment [11]. Table 1 presents basic information about sludge management methods together with the preceding unit processes as well as their advantages and disadvantages. Main groups of pollutants are also presented.

**Table 1:** Sewage sludge management methods together with the preceding unit processes and the most important advantages and disadvantages [2, 5–7, 12–16]

Sludge management methods	Unit processes applied to excess sludge	Disadvantages of the method	Advantages of the method	Main groups of potentially hazardous pollutants
Use in agriculture	Stabilization using earthworms Composting and stabilization in ponds Incineration Phosphorus recovery	Many standards to be met A relatively long stabilization time if low-temperature processes are used	Possibility of managing all sludge Low energy expenditure and reduction in concentrations of heavy metals (if earthworm stabilization is used)	High organic carbon load Aromatic hydrocarbons Halogenated organic compounds Heavy metals
Growing plants not intended for human consumption or feeding animals	Stabilization using earthworms Composting and stabilization in ponds	Limited application A relatively long stabilization time	Requirements pertaining to the quality of materials are lower than in the case of other uses connected with growing plants	High organic carbon load Aromatic hydrocarbons Halogenated organic compounds Heavy metals
Remediation and adjustment of soil to specific needs	Stabilization using earthworms Composting and stabilization in ponds	This method is not recommended by the European Union A relatively long stabilization time	Broad application Possibility of managing all sludge	High organic carbon load Aromatic hydrocarbons Halogenated organic compounds Heavy metals Phosphorus
Use in the construction industry	Vitrification Incineration Cementing Drying and pellet production	Problems with obtaining high strength Very high energy demand in the case of vitrification Many standards to be met The possibility of releasing heavy metals or organic pollutants (depending on the process used)	Partial refund of costs Broad application Possibility of managing all sludge	Heavy metals Phosphorus Chlorinated species
Use in industry	Drying and pellet production Phosphorus recovery Recovery of rare metals	High investment costs High costs of unit processes Complicated processes	Partial refund of costs Recovery of precious materials	Heavy metals Phosphorus
Recovery of energy	Drying and pellet production Anaerobic stabilization with biogas recovery Conventional incineration and co-incineration	High investment costs Processes are cost-efficient with large amounts of excess sludge Anaerobic fermentation susceptible to process inhibitors	Partial refund of costs Generation of energy from renewable resources Fewer odours	Carbon dioxide
Sludge-based production of adsorbents and bio-oil	Pyrolytic thermal processing	High energy demand Narrow market Many kinds of waste to be managed	Partial refund of costs Management of the majority of old residues	Aromatic hydrocarbons Halogenated organic compounds
Fat recovery and processing	Sludge treatment	Incomplete management (only some raw materials) It is necessary to install a fat recovery system	Partial refund of costs Low investment expenditures	Aromatic hydrocarbons Halogenated organic compounds Heavy metals
Storage at treatment plants and in landfills	Disinfection and chemical stabilization Incineration Vitrification Solidification of materials	This method is not recommended by the European Union Incomplete management Incurred management costs are not recovered	Simple methods Less restrictive standards as compared to other methods	High organic carbon load Aromatic hydrocarbons Heavy metals Phosphorus Halogenated organic compounds Chlorinated species

## Legal acts concerning the management of processed sewage sludge

Due to the complexity of the waste management problem, the number of legal acts pertaining to sewage sludge processing is vast. Basic legal regulations in the EU concerning the disposal of sewage waste are included in Council Directive 86/278/EEC of 12 June 1986. The Directive applies to requirements that sewage sludge must meet in the case of agricultural use. Council Directive 91/271/EEC adopted on 21 May 1991, amended by Commission Directive 98/15/EC, contained in the Water Framework Directive, concerns the collection, treatment and discharge of municipal wastewater as well as treatment and discharge of wastewater from certain industrial sectors. It also obliges monitoring of wastewater treatment process and the quality of obtained sewage sludge. The directive also prohibits disposal of sludge to surface waters. In addition, it introduces detailed monitoring requirements and forces EU Member States to submit reports on their sludge disposal activities every 2 years. According to the presented directive, sewage sludge should be reused, which has led to constant development of new sewage sludge management and reuse methods. Moreover, the Water Framework Directive treats sludge as a new product/substrate rather than as waste. Another directive related to sewage sludge is Council Directive 1999/31/EC of 26 April 1999 on the landfill of waste (Landfill Directive), which obliges EU Member States to limit the storage of biodegradable municipal waste. According to the European Parliament and Council Directive 2008/98/EC of 19 November 2008 on waste, the main priority is to prevent waste (including sewage sludge) or to prepare it for reuse, recycling or other forms of recovery.

The EU directives for sewage sludge and its management are based mainly on a warning system. In addition, they set many limits and restrictions when sewage sludge is, for example, managed in agriculture. These stipulations are generally more stringent than, for instance, the ones presented by U.S. EPA. The most important of the above-mentioned directives is the Landfill Directive, where the limitations of the amount of sewage sludge and other organic waste on landfills are presented. The slow but constant rise in the number of households connected to sewer networks and the increase in the level of wastewater treatment and of implementation of the directive, have contributed to the increase in the amount of sludge that must be managed. In addition, member states, in accordance with the accepted commitments, introduce their own legal acts which are often stricter than the EU legislation. Necessity of raw materials and energy recovery is growing together with the interest in environmental protection, so new legal acts pertaining to this subject are still being created. Many of them define the maximum allowable concentrations of pollutants in the managed sludge, which can vary depending on the adopted management method. EU directives which pertain to waste management methods and the definition of the maximum allowable concentrations are implemented in each country which joins the European Union [2, 17], owing to which emission standards are harmonized within the EU [5]. As a result, some national regulations are repealed, which may occasionally result in creation of legal gaps regarding regulations pertaining to specific standards for a given type of pollutants [14, 15]. The limit values for pollutants in sludge in the EU are restricted to several substances or groups of substances. In 2000 the European Commission proposed limit values for the so-called sum of absorbable organohalogens (AOX) and other organic contaminants such as: linear alkylbenzene sulfonates (LAS), di(2-ethylhexyl)phthalate (DEHP), nonylphenole and nonylphenole ethoxylates (NP/NPE), polyromantic hydrocarbons (PAH), polychlorinated biphenyls (PCB) and PCDD/F but hitherto, no legislation has been implemented. This is mainly due to lack of data on concentration of organic pollutants in sewage sludge. Moreover, a uniform approach to organic pollution has not been implemented yet [14, 18]. Suciu et al. [14] demonstrated in a 4-year study that the typical PAH content in sewage sludge used as a fertilizer does not exceed EU standards (6 mg/kg PAHs), which means that it can be used without the hazard of potential environmental pollution.

Other countries, such as the UK, USA and Canada, claim that typical concentrations of organic pollutants in sewage sludge are not dangerous to soil quality, human health, or the environment [19].

**Fig. 1:**
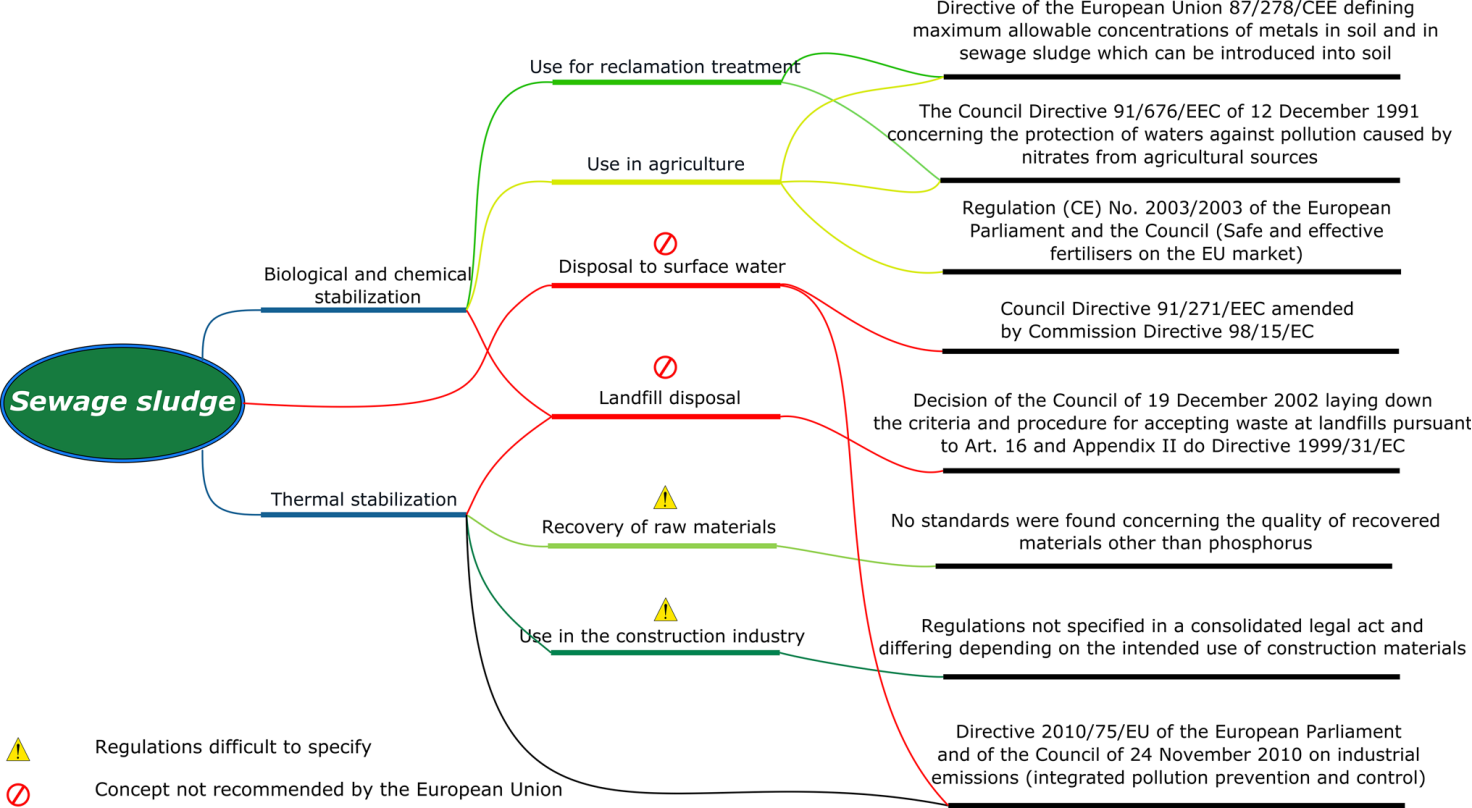
Legal regulations connected with selected sewage sludge management methods [5, 16, 17, 20, 21]

It should be remembered that each material introduced into the environment must not be a threat to fauna and flora. Even if pollutant emission standards are not specified, it is necessary to control pollutants released into the environment. Characteristics of main legal acts connected with specific sewage sludge management methods are presented in Fig. 1.

Another group of legal acts includes those concerning the obligation to monitor the condition of the environment, into which these pollutants are introduced. Information contained in them influences the requirements pertaining to the quality of pro-ecologically managed waste introduced into the environment. These legal acts include:

- Directive 2000/60/EC of the European Parliament and of the Council of 23 October 2000 establishing a framework for Community action in the field of water policy.
- Council Directive 86/278/EEC of 12 June 1986 on the protection of the environment, and in particular of the soil, when sewage sludge is used in agriculture.
- Council Directive 91/676/EEC of 12 December 1991 concerning the protection of waters against pollution caused by nitrates from agricultural sources.
- Council Directive 91/271/EEC of 21 May 1991 concerning waste water treatment.
- Directive 2008/98/EC of the European Parliament and of the Council of 19 November 2008 on waste and repealing certain directives.
- Council Directive 1999/31/EC of 26 April 1999 on the landfill of waste.
- Directive of the European Parliament and the Council 2008/50/EC of 21 May 2008 on ambient air quality and cleaner air for Europe.
- Directive 2004/35/CE of the European Parliament and of the Council of 21 April 2004 on environmental liability with regard to the prevention and remedying of environmental damage.
- Directive 2010/75/EU of the European Parliament and of the Council of 24 November 2010 on industrial emissions (integrated pollution prevention and control).

## Analytical methods used for the development of sewage sludge management technologies

**Fig. 2:**
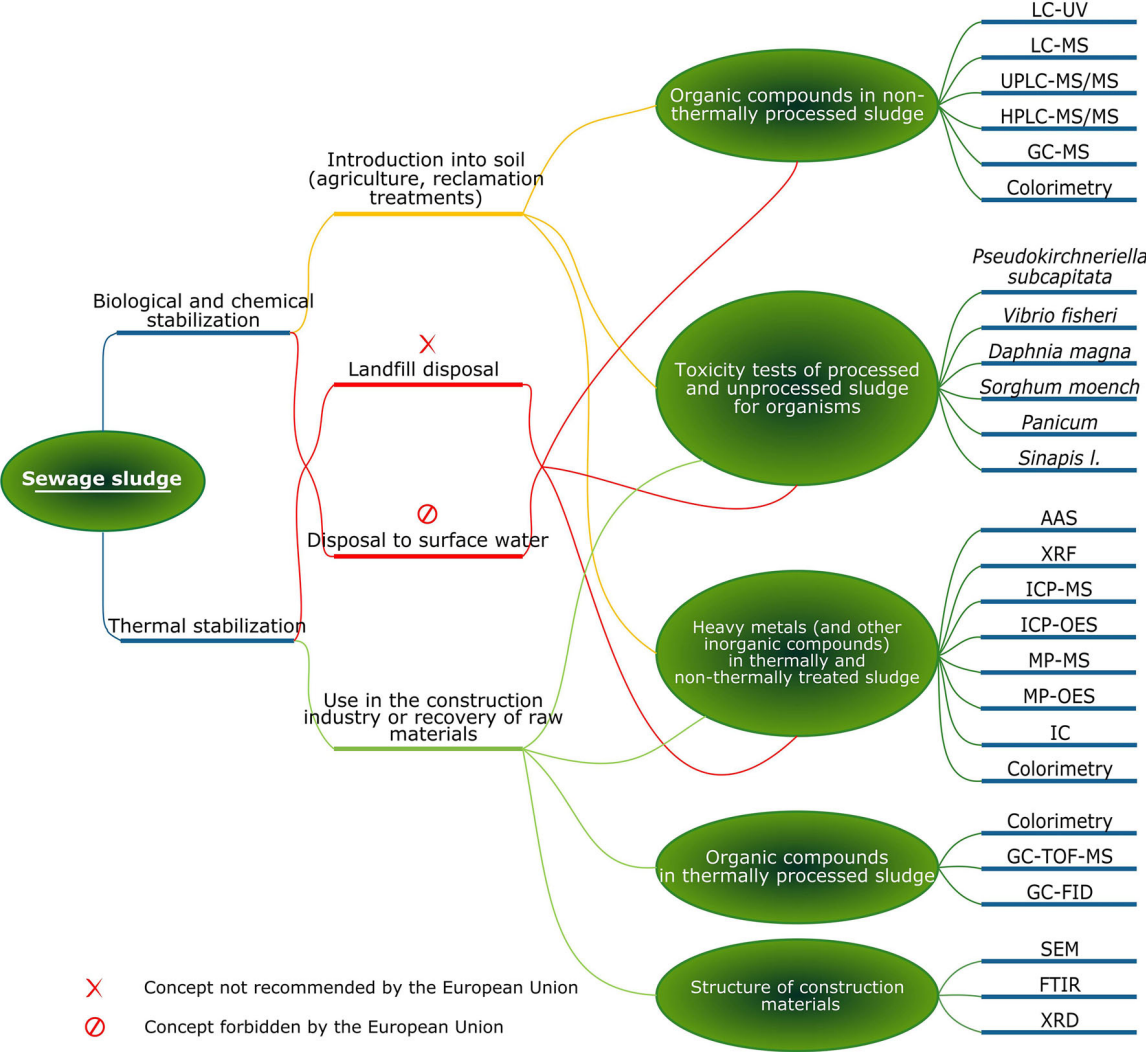
Analytical techniques used for the development of sewage sludge management methods [16, 22–27]

Sewage sludge can differ at every step of processing. Concentrations of main contaminants will change depending on the implemented technology. Some pollutants could even be totally neutralized during thermal treatment, however other may become more dangerous for the environment, for example due to their increased concentration. To develop an ecological method of waste management which, at the same time, meets all legal requirements, chemical analysis is a necessary auxiliary tool. Analytical techniques used for the development and further monitoring of management methods are simplified and presented as a diagram in Fig. 2.

### Organic species determination techniques

Sewage sludge treatment plants collect wastewater from many different agglomerations. Those could be densely or sparsely populated, heavily or lightly industrialized. A developed pharmaceutical industry may be present in one, while textile industry may be expanding in another. That is why sewage sludge from each wastewater treatment plant may differ significantly, also due to the concentration and variety of organic pollutants. Most organic species which may occur in sludge can be identified using chromatographic techniques. Among those species are pharmaceuticals, personal care products, steroid sex hormones, illicit drugs, benzotriazole and benzotriazole derivatives, volatile fatty acids, phenyl alkane acids, PAH, derivatives of benzoic acid, flame retardants, and many other compounds (e.g. cholest-4-ene, 3-methylphenol, aldehydes, esters, and pyrolysis residues). Pharmaceuticals and personal care products are also called “emerging organic contaminants” [10, 24, 28–33]. Concentrations of pharmaceuticals in wastewater are generally in the range of 1–0.001 µg/dm^3^ and their presence in sewage sludge and processed wastewater may be a potential hazard for the environment [34]. Nowadays, the most popular chromatographic methods for analysis of such pollutants in sewage sludge are gas chromatography–mass spectrometry (GC–MS), GC–MS/MS, and high performance liquid chromatography–mass spectrometry (HPLC–MS) [35, 36]. Moreover, it is worth mentioning that with the use of SPE and liquid chromatography–tandem mass spectrometry (LC–MS/MS) it is possible to determine a vast variety of antibiotics (ciprofloxacin, azithromycin, clarithromycin), analgesics (diclofenac), antithrombotics (dipyridamole), antifungals (fluconazole, clotrimazole), antihistaminics (fexofenadine, meclozine), cardiovascular drugs (eprosartan, telmisartan, valsartan), and psychostimulants (tramadol, carbamazepine) in influent wastewater during one analytical procedure. Moreover, the range of analysis, so the lowest and the highest determinable concentrations of abovementioned individuals (2 µg/dm^3^ of clotrimazole to 2270 µg/dm^3^ of telmisartan) can be considered as satisfactorily wide [34]. The same analytical technique was used for the analysis of 30 psychoactive pharmaceuticals, illegal drugs and their metabolites 3,4-methylenedioxymethamphetamine (MDMA), 3,4-methylenedioxyamphetamine (MDA), methylenedioxyethylamphetamine (MDEA), *N*-methyl-1,3-benzodioxolylbutanamine (MBDB), cotinine, methamphetamine, amphetamine, 11-nor-9-carboxy-Δ9-tetrahydrocannabinol (THC-COOH), cocaine, benzoylecgonine, cathinone, codeine, risperidone, oxycodone, 6-acetylmorphine, lysergic acid diethylamide (LSD), 2-oxy-3-hydroxy-LSD (OH-LSD), ketamine, norketamine, mephedrone, methylphenidate, tramadol, midazolam, venlafaxine, oxazepam, citalopram, buprenorphine, norbuprenorphine, 2-ethylidene-1,5-dimethyl-3,3-diphenylpyrrolidine (EDDP, methadone) in hospital effluents and wastewater after treatment [37]. Many other authors have reported the use of the mentioned SPE LC–MS/MS [38, 39] and that is why the technique may be considered as one of the most suitable for sewage sludge analysis.

Also GC–MS systems, frequently used during sewage sludge analysis, may be used for determination of many other organic pollutants in sewage sludge, such as alkylphenol ethoxylates, brominated flame retardants, biocides, and volatile nitrosamines [14, 40, 41]. In some cases GC–MS can also be used for monitoring of organic pollutants in soil. It is especially important if sewage sludge is introduced into it as a reclamation or fertilizing agent. Sixteen PAHs can be determined with the use of the mentioned technique: benzo[*g*,*h*,*i*]perylene, anthracene, fluorene, acenaphthene, phenanthrene, acenaphthylene, pyrene, benzo[*a*]anthracene, benzo[*b*]fluoranthene, chrysene, benzo[*k*]fluoranthene, fluoranthene, benzo[*a*]pyrene, indeno[1,2,3-*cd*]pyrene, dibenzo[*a*,*h*]anthracene, naphthalene [42]. Other techniques, such as gas chromatography–flame ionisation detector (GC–FID), may be used for determination of organochlorine pesticides, chlorobenzene and polychlorobiphenyl [43]. LC–MS is also used for detection of benzotriazole and surfactants [33, 44]. For other pharmaceutics and insecticides, also LC-UV can be used, which is not so expensive [45, 46].

It is worth mentioning that techniques of tandem mass spectrometry coupled with GC or LC, despite relatively high purchase costs, are widely adapted due to an extensive range of application. Those techniques provide high-performance separation, good selectivity and sensitivity, and thus are frequently used in environmental studies for determination of pharmaceuticals, personal care products or certain persistent organic pollutants. They can be used for the determination of organic substances in sludge processed at low temperatures, in residues formed after its thermal processing, and in many products that could be obtained during processing and management (e.g. bio-oils). Procedures for simultaneous determination of a wide variety of organic compounds are commonly implemented, as reported by Chokwe et al., Diaz-Cruz et al., and Herrero et al. [30, 32, 33]. In case of the determination of some organic compounds, HPLC or ultra performance liquid chromatography (UPLC), coupled with MS or MS/MS, can be used because a lot of compounds in sludge have a wide range of physicochemical properties and include many polar and non-volatile substances. These techniques are the most suitable because of their versatility, specificity, and selectivity [39, 47–49]. For the determination of carbohydrates or volatile fatty acids (VFA), a quick and easy colorimetric analysis is used. Owing to the heterogeneous structure of samples, composition of various kinds of polysaccharides and diverse responses to this analytical method (which may by under- or over-estimated), it is being replaced by more modern analytical techniques like for example previously mentioned GC–MS or HPLC [50–52]. It is important to mention that a variety of preparation techniques are crucial for proper execution of the mentioned analyses (vide infra).

### Inorganic species determination and other parameters analysis

Determination of inorganic species in wastewater, sewage sludge or ashes and dust obtained during thermal treatment is crucial for understanding and proper development of new management or treatment methods. As an example, the determination of NH_2_OH in treated wastewater helps to understand the process of N_2_O production and the quantitative relationship between the emission of those two compounds. For the analysis, spectrophotometric method with the use of ferric ammonium sulfate and 1,10-phenanthroline as the oxidation and chromogenic agent was used. The method is considered accurate if the concentration of hydroxylamine is higher than 0.05 mg/dm^3^ and the concentration of NO_2_^−^ is lower than 15.00 mg/dm^3^. However, PO_4_^3−^ can influence the measurement, however numerical correction methods proposed by the authors are described in the study [53]. In this and many other examples, the determination of certain anions is crucial for the implementation of proper analytical methods, or even for the control of treatment parameters. Moreover, the level of anion emissions is subject to inspection in many stages of sewage sludge management: landfilling of raw sewage sludge and solid residues obtained during the sewage sludge thermal treatment process, assessment of environmental friendliness of construction materials produced from wastes formed in sewage sludge treatment plants, or even in fertilizers produced from processed sewage sludge. To determine certain anion emissions levels, such as F^−^, Cl^−^, Br^−^, SO_4_^2−^, NO_3_^−^, PO_4_^3−^ etc., ion chromatography (IC) is employed in majority of studies [24, 54, 55]. Although, there are problems that could be solved with the use of alternative methods. For example, water soluble chloride ion content can be determined with the use of CNS 13407 Standard Method. This approach gives us a possibility to assess the environmental hazard connected with using sewage sludge ash as an additive for the production of low strength construction materials [56]. While designing management methods connected with the production of construction materials, it may be necessary to define the structure of the surface of the tested material. In this case, techniques such as scanning electron microscopy (SEM) would be suitable. Furthermore, Fourier-transform infrared spectroscopy (FTIR) gives the possibility to obtain information about the main functional groups, which it especially important if construction material was created with the use of dewatered sewage sludge and may contain significant amounts of organic impurities. X-ray analysis could also be used to determine mineral phases. Due to all these facts, the mentioned techniques help in assessing the quality of obtained product [10, 24, 57]. Analytical techniques can also be used to define the process parameters connected with specific management methods, e.g. thermogravimetry can be used to define the efficiency of incineration, depending on the composition of the analysed agent [58].

Determination of heavy metals and other elemental species is becoming more and more important at different stages of sewage sludge processing and management. Even without the necessity of their determination for monitoring the wastewater treatment process, heavy metals would have to be determined in the sewage sludge which is going to be used as soil amendments or fertilizers. Spectroscopic techniques such as atomic absorption spectroscopy (AAS) [1, 23, 59], X-ray diffraction (XRF), or even simple spectrophotometric determination of phosphorus using the molybdenum blue colorimetric standard [60, 61], can be used to determine analytes in extracts and directly in the processed materials. Nowadays AAS is one of the cheapest and therefore still one of the most frequently used techniques for metal determination in sewage sludge and processed sewage sludge [57, 59, 62]. X-ray analysis techniques are mostly employed for the determination of heavy metals in construction materials and sewage sludge ashes used for their production. These could be also used for quality assessment of raw materials before production of fertilizers from sewage sludge and sewage sludge ashes [63–66]. However, more and more often, modern analytical techniques, such as inductively coupled plasma-mass spectrometry (ICP-MS) [24, 67] or inductively coupled plasma-optical emission spectrometry (ICP-OES) [29, 64], are employed in such analyses. Apart from determination of heavy metals in managed sewage sludge, ashes or other process products, those techniques could be used for example to estimate the Fe, Cr and Ni concentration in effluents after the electro-Fenton oxidation process. In case of using such an alternative method of wastewater treatment, it is crucial to estimate the corrosion of electrodes and the environmental hazard connected with the emission of the mentioned metals to the environment [68]. It is worth mentioning that microwave plasma-mass spectrometry (MP-MS) or even microwave plasma-optical emission spectrometry (MP-OES) could be almost as efficient but cheaper alternative to such sophisticated analyses. The most important disadvantage of using the aforementioned techniques is the necessity of proper mineralization of the obtained sample. In some cases, pseudo total metal content could be sufficient [57], however in majority of cases the use of microwave assisted mineralization with the use of aqua regia or another strong mineralization agent would have to be used [62].

Often, the only way of developing a safe method for waste management involves controlling process parameters in order to learn if the end product meets the emission requirements. In tests of batches of products produced under various conditions, analytical methods belonging to the so-called screening methods can be useful, in which the measured signal is the cumulative parameter, e.g. total carbon, total oxygen, toxicity etc. For separate non-metallic element analysis, elemental analysers with the ability to measure total carbon, oxygen, nitrogen and hydrogen are often used [69, 70]. In some cases, parameters like total phosphorus can be measured with the use of the aforementioned methods such as AAS with previous mineralization. A proper mineralization technique can also make it possible to perform speciation analysis with the use of the same equipment [69, 70]. Such an approach will be discussed in a later chapter.

Bio-tests can be used for toxicity assessment. Here, a variety of parameters can be measured, such as effective dose or concentration, lethal dose or concentration, inhibition dose or concentration, etc. When performing bio-tests, the parameters measured could be mortality, growth, or reproduction factor of tested organisms. Tests are carried out by exposing the tested organisms to toxic substances under controlled conditions [71, 72]. In this way, it is possible to quickly exclude products which do not meet ecological requirements. The most frequently used toxicity tests used include tests of various microorganisms, such as: *Vibrio fisheri*, *Pseudokirchneriella subcapitata*; crustaceans, such as *Daphnia magna,* or plants, such as *Sorghum moench, Panicum* and *Sinapis* L. [23, 59, 72]. Commercial toxicity tests, such as *Daphtoxkit F magna* and *Microtox* are available on the market. Other organisms used during toxicity test are presented by Farre and Barceló [71]. However, such tests have one significant disadvantage. Toxicity analysis and assessment often last long, from few days to several weeks, since the response of a tested organism cannot be instantaneous in majority of cases. Therefore, more and more bio-tests are being created since every variable in conditions (time of exposure, organism used, different standard solutions) can give us a variety of new information.

## Preparation techniques

From an analytical point of view, sewage sludge is characterized by a complex matrix due to the heterogeneity of the composition, which is dependent on the type of sewage brought to a treatment plant. Sewage sludge also contains compounds that can interfere with the analysis of substances of interest. These compounds are present in wastewater (lipids, proteins etc.) or are introduced during its treatment (surfactants, iron chloride, polymeric colloids and lime). It is important to remove interferents from the samples during the preparation stage by means of an appropriate purification procedure [33]. It is especially important that the analytical method should be optimized in a way that will ensure limited use of toxic solvents and reduce the analysis time as much as possible. Using proper extraction techniques, such as pressurized liquid extraction (PLE) [31, 73], liquid–liquid extraction (LLE) [41], ultrasonic extraction (USE) [11, 73], microwave-assisted extraction (MAE) [73], accelerated solvent extraction (ASE) [11], SPE [11, 74], or solid phase microextraction (SPME) [41], is equally important. These sample preparation methods are usually multi-step and time and labour consuming, but they are necessary for proper analysis. It is not the aim of this publication to characterise every single extraction type, as these are described in previously cited articles. In the case of sample preparation for the determination of antibiotics and their derivatives (e.g. quinolone), the following environmentally friendly extraction techniques are used: USE, MAE, and PLE. MAE and PLE are the best extraction techniques because of their higher extraction yields, easy operation, shorter analysis times, and a high automation degree. PLE also provides good recoveries and adequate precision, thus it can also be used for the extraction of antiinflammatories, analgesics, antibiotics parabens, compounds called UV filters, musk fragrances and antimicrobials. Although USE is a simpler technique requiring the use of similar volumes of solvents, it is more tedious and requires much longer analysis times [31, 73]. Analysis of drug residues and their metabolites is not done routinely, but if it is performed, the extraction method used may also be ASE [11].

Sewage samples are a complex matrix due to the high content of organic matter and suspended solids. Where wastewater is highly hydrated, it is also possible to successfully use SPME, dispersive liquid–liquid microextraction (DLLME), or stir-bar sorptive extraction (SBSE). Before applying any of these techniques, sample filtration is essential to prevent clogging on the SPE cartridge and/or to remove the particulate matter from the aqueous phase. In the analysis of sewage sludge with the low moisture content, preparation techniques for solid samples can be used. It is possible to use ultrasound-assisted solvent extraction (USAE) and liquid–solid extraction (LSE), based on quick, easy, cheap, effective, rugged, and safe (QuEChERS) extraction or conventional methods, such as shaking. Since most of these extraction techniques use organic solvents, an evaporation step after extraction is essential if the sample is analyzed using LC or GC. Moreover, SPE is the most effective clean up method of extracts of sludge samples containing e.g. pharmaceutical residues. Prior to using these extraction techniques, samples are usually dehydrated or freeze-dried, and then crushed and sieved to obtain homogenous particles [32, 33].

Turbulent flow chromatography, being a modern technique, allows us to separate the analytes of interest from complex matrices in an online system, minimizing the sample preparation time and limiting the use of toxic solvents. Using this approach, the total analysis time is reduced from 3 h to about 50 min [38]. Owing to the use of capillary chromatographic columns, it is possible to reduce the use of the frequently toxic mobile phase even more than tenfold. In the case of some measurements, e.g. the determination of free cyanides and cyanates, also less complicated and less expensive spectrophotometric techniques can be used (chloramine and pyridine-barbituric acid) [23].

If sewage sludge thermal utilization methods are implemented, the rising concentration of heavy metals will become a very important issue from the ecological point of view. If they are not leached from the processed sludge, it can be safely used as construction materials [16]. As mentioned before, if a management method assumes the introduction of sludge into the soil as fertilizer, the heavy metal ion content should be closely monitored. It is very important to understand the behaviour of certain pollutants in the environment, their bioavailability and amount that is extracted under certain circumstances. All kinds of extraction techniques can be used for this purpose as they allow to estimate the degree of leaching of potentially hazardous elements from the managed sludge into the environment [15, 75]. Possible compositions of solutions which can be used for the extraction of various fractions of trace metals from soils and sludge are presented in Table 2.

**Table 2:** Most frequently used solutions applied for the extraction of various fractions of trace elements from the soil [62, 67, 69, 70, 76–78]

Fractions of trace elements	The composition of the aqueous extraction solution together with the concentration of the agent used/mol/dm^3^
Metals occurring in the soil solution	Distilled/deionised water CaCl_2_ (0.01)
Easily replaceable metals	CH_3_COONH_4_ (0.5) MgSO_4_ (0.2) NH_4_Cl (0.1) NH_4_NO_3_ (1.0) MgCl_2_ (1.0) CH_3_COOH (0.1)
Bound to carbonates	CH_3_COONa (1.0), pH 5 CH_3_COOH
Fe–Mn oxides bound	NH_2_OH·HCl in 25% CH_3_COOH
Adsorbed metals	CH_3_COOH (0.5) HCl (0.1) HNO_3_ (0.1)
Metals bonded with organic matter	Ethylenediaminetetraacetic acid (EDTA) (0.05) Ethylenediamine bis(2-hydroxyphenyl)acetic acid (EDDHA) (0.05) Pentetic acid (DTPA) (0.005) + triethylamine (TEA) (0.1) + CaCl_2_ (0.01) CH_3_COONH_4_ (3.2) in 25% HNO_3_ H_2_O_2_ (8.8) in (0.02) HNO_3_
Metals connected with hydrated oxides	CH_3_COONH_4_ (1.0) + C_6_H_6_O_2_ (0.002) (COONH_4_)_2_ (0.2) + (COOH)_2_ (0.15), pH 3.3
Metals connected with aluminosilicates (residues)	HF Decomposition with various fluxes Aqua regia HNO_3_ + HClO_4_ + H_2_O_2_
Metals in sewage sludge and adsorbed on the surface of ashes	HF + HNO_3_ + HClO_4_ (concentrated) HNO_3_ + H_2_SO_4_ (concentrated)
Total metals concentration in soil	HNO_3_ + HClO_4_ + H_2_O_2_ HNO_3_ + HClO_4_ + H_2_SO_4_
Phosphorus	HNO_3_ + HClO_4_ + H_2_O_2_

## Conclusions

Sludge managed without high-temperature processing may contain a very broad range of potentially hazardous organic compounds, the determination and inspection of which will involve high financial expenditures. High-temperature methods neutralize even 99% of potentially hazardous organic substances. However, after thermal processing, the concentration of heavy metal ions increases. Still, the quantity of heavy metals introduced into the environment will be the same as for low-temperature processing. It does not mean that the amount of released ions will be the same. In both cases, it is important that heavy metal ions should be immobilized in managed materials or converted into a harmless form, which is possible in the case of using high-temperature processes or cementation/stabilization technologies.

In many cases, specific chemical species determination during the implementation of a sewage sludge management method is mandatory due to the law. For this reason, chemical analysis may be an auxiliary tool at the process line design stage, and also a necessary element at further stages of inspection and monitoring of individual elements of the environment, in particular in view of the fact that storage of the managed sewage sludge still cannot be totally avoided. Many processes have not been thoroughly explored yet, for example using sand collected in wastewater treatment plants as a binder during the production of construction materials as part of a sewage sludge management method. It is necessary to perform tests and analyses on a laboratory and technical scale to prove their usefulness.

The range of pollutants found in processed sewage sludge and other materials received during processing reflects what is being used in society and fed into the sewage system by industry. The behaviour of emerging contaminants in processed sewage sludge demands more investigation, since the available data only give a very general overview. At present, there are several advanced analytical methods for detecting and quantifying emerging contaminants, and new ones are appearing. In the majority of cases, SPE–LC–MS systems are used for organic species determination. “Multidimensional” or “tandem” systems like GCxGC–MS or LC–MS/MS will be more frequently used for sludge analysis in the future. For inorganic species determination, AAS based techniques are most often used, but ICP-MS and ICP-OES are becoming more and more popular due to broader application capabilities. Cheaper techniques, based on microwave plasma generation, such as MP-MS or MP-OES, will probably gain popularity in the nearest future. Still, few analytical methods are in everyday use for quality assessment of processed sewage sludge and other materials received during processing. Constant development of new analytical techniques which could be of use in sewage sludge treatment plants is inevitable and essential for developing ecological sewage sludge processing and management methods.
